# Standardization and advancements efforts in breast diffusion-weighted imaging

**DOI:** 10.1007/s11604-024-01696-z

**Published:** 2024-12-06

**Authors:** Mami Iima, Maya Honda, Hiroko Satake, Masako Kataoka

**Affiliations:** 1 Department of Fundamental Development for Advanced Low Invasive Diagnostic Imaging, Nagoya University Graduate School of Medicine, 65 Tsurumai-cho, Showa-ku, Nagoya, Aichi 466-8550 Japan; 2 Department of Diagnostic Imaging and Nuclear Medicine, Graduate School of Medicine, Kyoto University, Kyoto, Kyoto Japan; 3 Department of Diagnostic Radiology, Kansai Electric Power Hospital, Osaka, Osaka Japan; 4 Department of Radiology, Nagoya University Graduate School of Medicine, Nagoya, Aichi Japan

**Keywords:** Magnetic resonance imaging, Diffusion-weighted imaging, Breast cancer, Apparent diffusion coefficient

## Abstract

Recent advancements in breast magnetic resonance imaging (MRI) have significantly enhanced breast cancer detection and characterization. Breast MRI offers superior sensitivity, particularly valuable for high-risk screening and assessing disease extent. Abbreviated protocols have emerged, providing efficient cancer detection while reducing scan time and cost. Diffusion-weighted imaging (DWI), a non-contrast technique, has shown promise in differentiating malignant from benign lesions. It offers shorter scanning times and eliminates contrast agent risks. Apparent diffusion coefficient (ADC) values provide quantitative measures for lesion characterization, potentially reducing unnecessary biopsies. Studies have revealed some correlations between ADC values and hormone receptor status in breast cancers, although substantial variability exists among studies. However, standardization remains challenging. Initiatives such as European Society of Breast Imaging (EUSOBI), Diffusion-Weighted Imaging Screening Trial (DWIST), Quantitative Imaging Biomarkers Alliance (QIBA) have proposed guidelines to ensure consistency in imaging protocols and equipment specifications, addressing variability in ADC measurements across different sites and vendors. Advanced techniques like Intravoxel incoherent motion (IVIM) and non-Gaussian DWI offer insights into tissue microvasculature and microstructure. Despite ongoing challenges, the integration of these advanced MRI techniques shows great promise for improving breast cancer diagnosis, characterization, and treatment planning. Continued research and standardization efforts are crucial for maximizing the potential of breast DWI in enhancing patient care and outcomes.

## Introduction

Breast MRI offers numerous advantages over traditional imaging techniques, making it an invaluable tool in various clinical scenarios [1]. Breast MRI is a powerful imaging technique with superior sensitivity in detecting breast cancers, particularly valuable for high-risk screening and assessing disease extent in diagnosed cases. It excels at identifying primary tumors in metastatic scenarios, evaluating pathologic nipple discharge, characterizing lesions, and assessing breast implant integrity, making it an essential tool in comprehensive breast care [2]. Recent advancements have led to the development of abbreviated breast MRI protocols, offering a streamlined approach to cancer detection while reducing scan time and cost [3].

Within the realm of breast MRI, diffusion-weighted imaging (DWI) has gained significant attention as a promising technique for breast cancer diagnostics. DWI offers several notable advantages over traditional methods. As a non-contrast technique, it eliminates the need for contrast agents, reducing potential risks to patients and making it suitable for those with contraindications to contrast media. The efficiency of DWI translates to shorter scanning times, enhancing patient comfort [4]. Furthermore, DWI provides quantitative measures through apparent diffusion coefficient (ADC) values, playing a crucial role in differentiating between various types of lesions.

Various studies have supported the effectiveness of DWI in breast cancer diagnosis, demonstrating sensitivity and specificity comparable to dynamic contrast-enhanced MRI (DCE-MRI). When used in conjunction with DCE-MRI, the use of ADC thresholds has shown promise in reducing unnecessary biopsies [5, 6].

Despite its advantages, standardization remains a challenge in breast DWI. Various organizations have developed guidelines and protocols to ensure consistency and reliability across different institutions and studies. The selection of appropriate b-values is crucial for accurate ADC measurements. Recent research has also shown that breast density affects DWI image quality, suggesting a need for optimization across different breast types [7].

Advanced DWI techniques, such as intravoxel incoherent motion (IVIM) and diffusion kurtosis imaging (DKI), offer additional insights into tissue microstructure and microvasculature, further enhancing its diagnostic capabilities. These techniques provide more detailed information about tissue properties, potentially improving the accuracy of breast cancer characterization and treatment planning.

This paper aims to provide a comprehensive overview of the various advancements and standardization efforts in breast DWI. We will explore the latest techniques, discuss ongoing challenges, and examine the initiatives aimed at improving consistency and reliability in breast DWI across different clinical settings. By reviewing these developments, we hope to highlight the evolving role of DWI in breast cancer care.

## DWI quantitative measures and ADC thresholds in breast cancer diagnosis

One of the key strengths of breast DWI lies in its ability to provide good visibility and high specificity in distinguishing malignant lesions from benign ones. This is further enhanced by the quantitative evaluation made possible through ADC values, which play a crucial role in differentiating between various types of lesions [8]. When comparing the sensitivity and specificity of different imaging techniques, research has shown promising results for DWI. A study reported that while DCE-MRI demonstrated 100% sensitivity and 76.6% specificity, DWI achieved 82% sensitivity and 86.8% specificity [9]. A recent systematic review including 28 studies and 4406 lesions (1676 malignant, 2730 benign) in 3787 patients revealed that the pooled sensitivity and specificity of standalone DWI were 86.5% and 83.5%, and that when compared with DCE-MRI, standalone DWI had modestly lower sensitivity (88.9% vs 95.1%, *P* = 0.004) and similar specificity (82.0% vs 82.2%, *P* = 0.97 ) [10]. Notably, the combination of DCE-MRI and DWI yielded optimal results with 96.8% sensitivity and 83.8% specificity, highlighting the potential of integrating these techniques [9]. On the other hand, combination of DWI and T2WI is one promising non-contrast method. One study showed that a decision tree for these unenhanced sequences including lesion margin and ADC could distinguish malignant from benign lesions with AUCs of 0.77–0.87 [11]. The combination of ADC and Kaiser score may be also beneficial for less experienced radiologists [12].

The use of ADC thresholds has emerged as a valuable tool in breast cancer diagnosis, providing quantitative measures to differentiate malignant from benign lesions. The increased cellularity and hindered water diffusion in malignant tumors typically results in lower ADC values compared to benign lesions or normal tissue. Meta-analyses report ADC ranges of 0.8–1.3 × 10^-3^ mm^2^/s for malignant lesions, 1.2–2.0 × 10^-3^ mm^2^/s for benign lesions, and 1.7–2.0 × 10^-3^ mm^2^/s for normal breast tissue. While these ranges overlap somewhat, proposed ADC thresholds for distinguishing malignant from benign lesions generally fall between 1.0 and 1.6 × 10^-3^ mm^2^/s. However, optimal thresholds may vary depending on factors like MRI protocols and patient populations. Therefore, ADC measurements should be interpreted cautiously in conjunction with other imaging and clinical findings rather than used as a standalone diagnostic criterion [8]. These thresholds can also potentially reduce the number of unnecessary biopsies, as shown in multi-center studies [5, 6]. The ECOG-ACRIN trial found that an ADC threshold of 1.53 × 10^-3^ mm^2^/s (obtained with b-values of 0 and 800 s/mm^2^) could reduce unnecessary biopsies by 20.9% while maintaining 100% sensitivity for malignancies. Another multicenter study by Clauser et al. reported that an ADC threshold of 1.5 × 10^-3^ mm^2^/s could potentially avoid 46% of benign biopsies [5, 6]. A recent validation study suggests that applying the ADC cutoff can reduce biopsies by up to 16%, with the tradeoff being reduced sensitivity for in situ and microinvasive disease presenting as non-mass enhancement [13].Classification of breast lesions like Breast Imaging Reporting and Data System (BI-RADS) MRI based on ADC alone has also been considered, but has some limitations at this time [14–16].

**Fig. 1:**
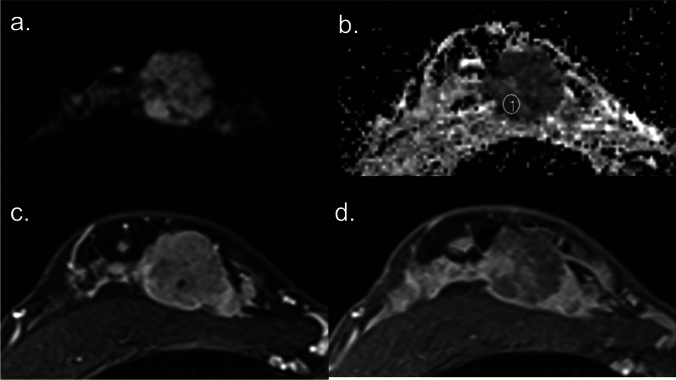
Diffusion-weighted (DW) image with b value = 1000 sec/mm^2^ (**a**), corresponding apparent diffusion coefficient (ADC) map (**b**), early phase dynamic contrast-enhanced (DCE) image (**c**), and delayed phase DCE image (**d**) of breast cancer. The breast cancer exhibits hindered diffusion and hyperintense on the DW image (**a**) with a correspondingly low ADC value of approximately 0.77 × 10^-3^ mm^2^/s on the ADC map (**b**). The lesion demonstrates fast in the early phase (**c**) and washout enhancement in the delayed phase (**d**), characteristic of a malignant breast tumor.

**Fig.2:**
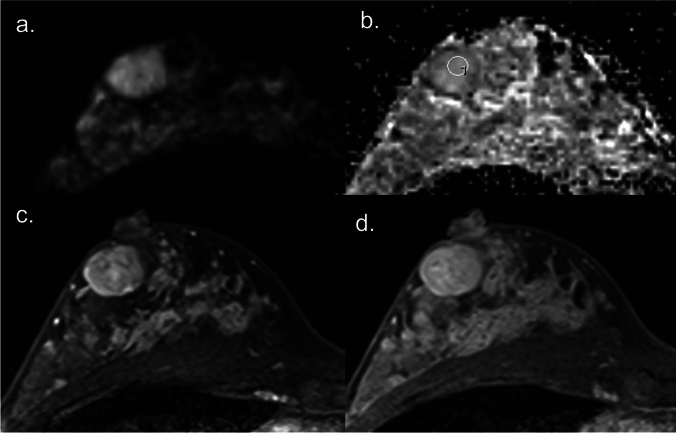
DW image with b value = 1000 sec/mm^2^ (**a**), corresponding ADC map (**b**), early phase DCE image (**c**), and delayed phase DCE image (**d**) of a fibroadenoma. The fibroadenoma shows hyperintense on the DW image (**a**) with a high ADC value of approximately 1.65 x 10^-3^ mm^2^/s on the ADC map (**b**). The lesion exhibits slow initial contrast enhancement in the early phase (**c**) and persistent enhancement in the delayed phase (**d**), typical of a benign breast lesion.

Generally, low ADC values are indicative of malignant lesions, while higher ADC values suggest benign lesions, as shown in Figs. 1 and 2. This is primarily due to higher cellularity and more restricted water diffusion in malignant tumors [17]. This quantitative approach adds an extra layer of confidence to the diagnostic process, potentially improving accuracy and patient outcomes.

## Correlation of ADC values with hormone receptor status

Meta-analyses of multiple studies have shown significant correlations between ADC values and hormone receptor status in breast cancers: Estrogen receptor (ER)-positive and progesterone receptor (PR)-positive cancers generally exhibit lower ADCs than their negative counterparts. Human epidermal growth factor receptor 2 (HER2)-positive cancers tend to have higher ADCs than HER2-negative cancers. Ki-67-positive cancers generally show lower ADCs than Ki-67-negative cancers [17]. However, there is considerable heterogeneity among studies, highlighting the need for standardization in research methodologies and acquisition protocols.

## Use of DWI for breast cancer treatment response

Neoadjuvant systemic treatment is often the choice for locally advanced or some subtypes of breast cancer. DWI reflects tumor cellularity and water diffusivity, and can detect tumor response to systemic treatment [18]. The American College of Radiology Imaging Network (ACRIN) 6698 trial was performed as a sub-study of the multicenter Investigation of Serial Studies to Predict Your Therapeutic Response with Imaging and Molecular Analysis 2 (I-SPY 2) trial to test the value of DWI as an early marker of breast cancer response to neoadjuvant systemic treatment [19]. This study evaluated in 242 women enrolled at 10 institutions and showed that the percentage change in tumor ADC at midtreatment (12 weeks) and after treatment were moderately predictive of pathologic complete response (pCR) with the area under the receiver operating characteristic curves (AUCs) of 0.60 and 0.61, respectively. More recently, the results from Breast Multiparametric MRI for prediction of neoadjuvant chemotherapy (BMMR2) challenge were published [20]. The BMMR2 challenge, using I-SPY 2/ACRIN 6698 trial data, evaluated pCR prediction models from breast MRI. Three teams surpassed the ACRIN 6698 benchmark AUC (0.78), achieving AUCs of 0.80–0.84, using varied approaches from feature extraction to AI, and incorporating DCE-MRI and DWI alone or combined.

Evaluation of enhancing tumor volume using contrast-enhanced MRI is also reported to predict the tumor response to neoadjuvant therapy, but is time-consuming. A recent study revealed DWI-maximum intensity projection could be an alternative to contrast-enhanced MRI for tumor volume assessment in a shorter processing time [21].

## Standardization efforts in breast DWI

Standardization efforts in breast DWI have become increasingly important as the technique gains wider adoption in clinical practice. Various initiatives, including EUSOBI [8], DWIST [22], and QIBA [23], have developed guidelines and protocols to ensure consistency and reliability in breast DWI across different institutions and studies.

The standardization guidelines typically specify key technical parameters. For instance, they recommend using magnetic field strengths of 1.5T or 3.0T with dedicated breast coils to ensure optimal image quality. The field of view should encompass both breasts, with the option to include the axillary region for comprehensive imaging. Slice thickness standards vary slightly but generally fall within the range of 3–5 mm.

**Fig. 3:**
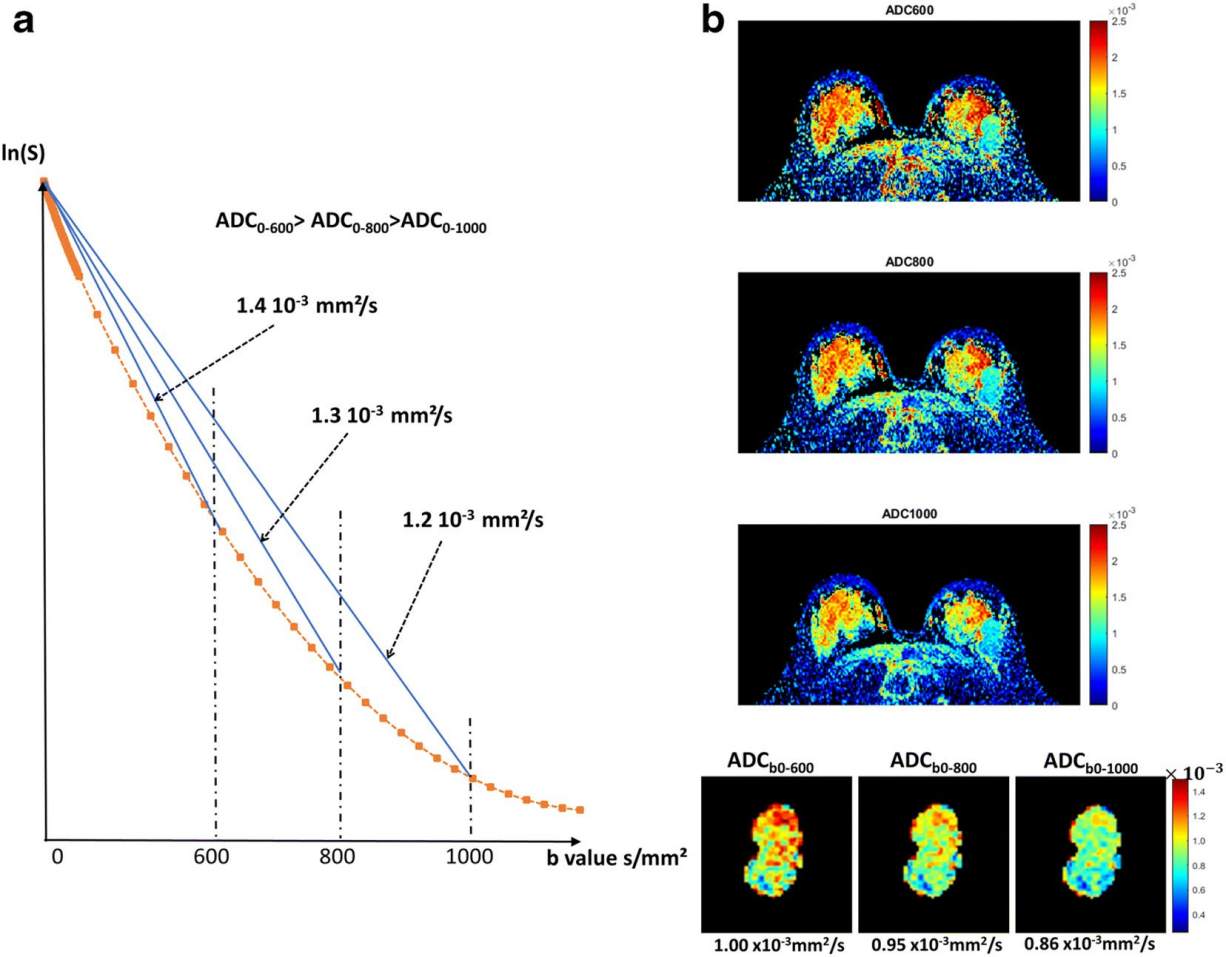
DWI signal decay with b value. **a** (left) Water diffusion in tissues results in a decrease of the MRI signal with the amount of diffusion weighting of the MRI sequence which is characterized by its b value. For pure water, the log of the signal decrease is a straight line. However, in tissues, diffusion becomes hindered by obstacles (e.g., cell membranes), and the attenuation becomes curved (so-called non-Gaussian diffusion). As a result, the ADC, which is the slope of the attenuation between b = 0 and another b value, decreases when this b value increases. **b** (right, top): Example of a breast tumor (light blue area in left breast outer part) exhibiting different ADC values and textures on ADC maps when using different b values (600, 800, and 1000 s/mm^2^); (right, bottom): Region of interest placed over the lesion showing the ADC decrease with b value (different ADC color scales have been used for the upper and lower parts). Figure adapted from [24.

Importantly, the selection of appropriate b-values is critical for accurate ADC measurements, as ADC is significantly affected by b values (Fig. 3).

The EUSOBI consensus guideline suggests 800 sec/mm^2^ [8], while DWIST recommends 800 sec/mm^2^ for characterization and 1500 sec/mm^2^ for detection [22]. The guidelines also emphasize the importance of effective fat suppression in breast DWI to avoid ADC underestimation and chemical shift artifacts. Spectral adiabatic inversion recovery (SPAIR) is recommended over short-tau inversion recovery (STIR), as STIR can lead to signal attenuation in all tissues, potentially causing ADC over- or underestimation [25].

These efforts aim to establish minimum requirements for equipment and imaging protocols, crucial for producing comparable results across institutions. However, challenges persist, including variability in ADC measurements across different sites and equipment vendors [26]. Reaching a consensus on protocol details remains a work in progress. Specifically, agreement on the optimal set of b-values and imaging techniques continues to evolve.

Variations in breast tissue and density affect DWI image quality. Recent research by Wielema M et al. shows that DWI image quality improves with increased breast density, suggesting its potential effectiveness for screening dense breasts while indicating a need for optimization in less dense breasts [7].

Previous research also provides encouraging evidence for the robustness of DWI in clinical practice. A meta-analysis of 61 studies, encompassing 4778 patients and 5205 lesions, demonstrates that DWI maintains high diagnostic performance in differentiating malignant from benign breast lesions, despite variations in acquisition protocols or scanner types. With an overall sensitivity of 90% and specificity of 86%, and no significant difference between 1.5T and 3.0T MRI units, DWI maintains effective in differentiating between benign and malignant breast lesions despite variations in acquisition protocols or scanner types [27]. These results are promising and suggest that while variability in ADC measurements across different sites and equipment can potentially affect lesion characterization, DWI proves effective in differentiating between benign and malignant breast lesions despite these variations. Nonetheless, continued collaboration and research in the field remain essential to further refine and standardize breast DWI techniques, ensuring even greater consistency and reliability in clinical practice.

## Qualitative evaluation methods

While quantitative methods like ADC measurements are common in breast DWI, qualitative approaches also play a role in lesion assessment. Qualitative evaluation involves visual interpretation of DW images and ADC maps, similar to the PI-RADS system used in prostate imaging [28]. Our previous investigation found that multiple b-value DWI (5b-value DWI) showed promise in breast lesion assessment with good observer agreement, but its diagnostic performance (sensitivity 79.5–84.6%, specificity 62.5–64.3%) remained inferior to combined MRI with dynamic contrast-enhanced images (sensitivity 97.4%, specificity 75.0–78.6%) [29].

In terms of lesion detection, although previous results examining breast cancer detection by DWI alone have been somewhat inconsistent in terms of sensitivity and specificity [30], a recent study examined how training affects radiologists' performance in interpreting unenhanced breast MRI with DWI for cancer detection [31]. Sixteen breast radiologists reviewed 96 breast scans before and after a 2-h training session. Results showed that brief training significantly improved specificity (90.8–95.2%) and accuracy (83.5% to 85.9%), while inter-reader agreement also increased. The study suggests that dedicated training can enhance radiologists' ability to interpret unenhanced breast MRI with DWI, indicating its potential as a standalone method for breast cancer detection. DWI also detected significantly more clinically occult contralateral breast cancers (23 out of 30, 76.7%) with fewer biopsy recommendations compared to combined mammography and ultrasound (12 out of 30, 40.0%) in women with newly diagnosed breast cancer, demonstrating a higher cancer detection rate (2.0% vs 1.0%) and positive predictive value for biopsy (42.1% vs 18.5%) [32]. Another recent study evaluated the impact of double reading of breast DWI in the detection of breast cancer [33]. This study comprised 378 women with 41% of high-risk screening/surveillance MRI. Two breast radiologists evaluated high- and low-b images and ADC maps, allowing quantitative evaluation on readers’ discretion. Each breast was labeled as positive/suspect or negative by each reader, and cases judged positive/suspect by one or more readers were judged positive. The results showed a sensitivity of 93% and a specificity of 86%, with a 71% sensitivity for cancers ≤ 10 mm, promising for the feasibility of DWI screening. Recent technical advancements such as simultaneous multi-slice echo-planar diffusion-weighted imaging (SMS-DWI) might also help to improve lesion conspicuity [34]. In addition, a large multi-center study assessing the screening ability of DWI is being conducted in Korea [22], and will serve as a strong evidence for DWI screening.

## Advanced techniques in diffusion-weighted imaging

**Fig. 4:**
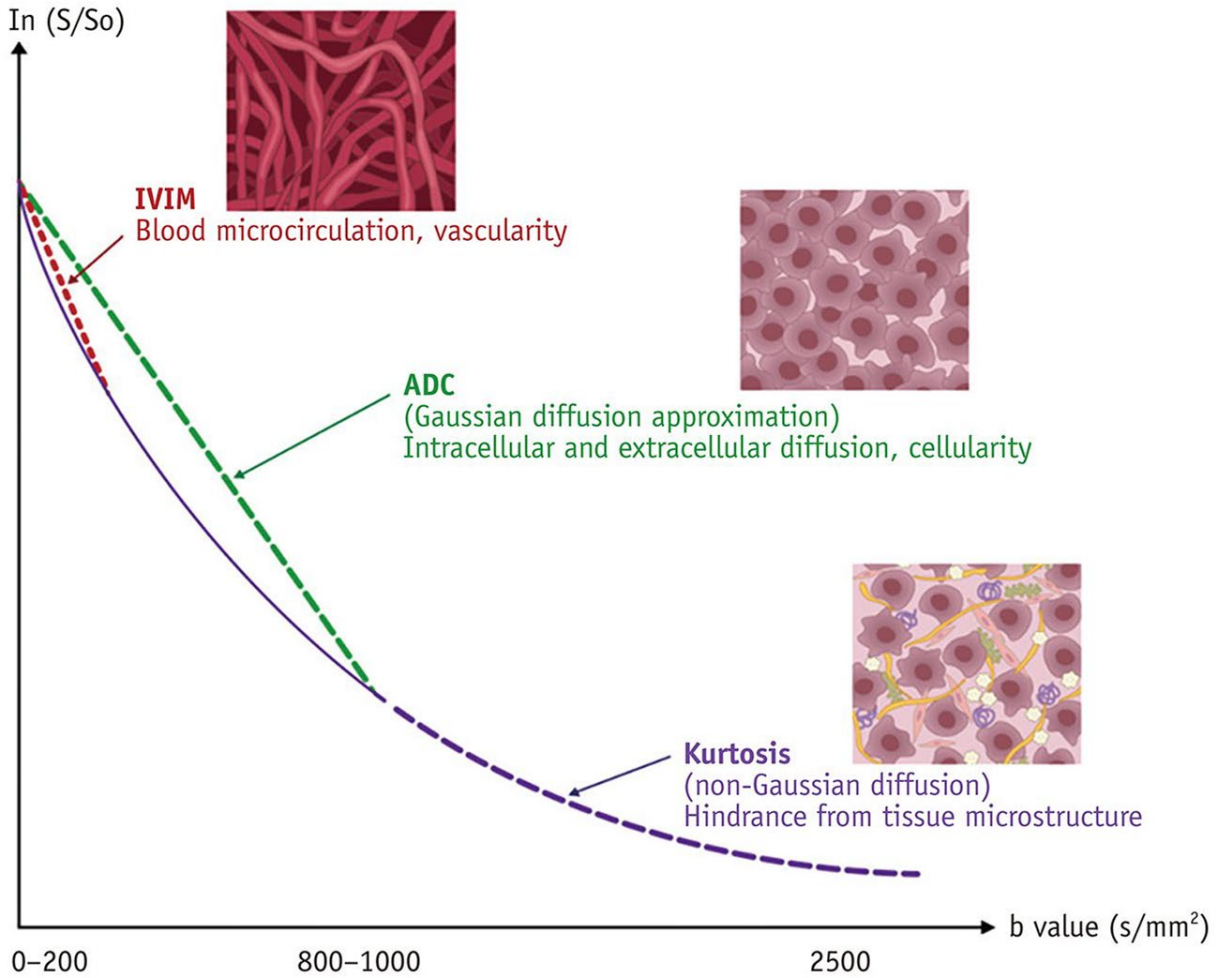
Example of a diffusion signal decay in typical breast lesions. IVIM offers insights into blood microcirculation and tissue vascularity, whereas the ADC reflects both intracellular and extracellular water diffusion, providing information about tissue cellularity. Kurtosis quantifies the deviation of water molecule diffusion from Gaussian distribution, offering information about hindrances caused by the tissue microstructure. IVIM = intravoxel incoherent motion Figure adapted from [17].

Beyond standard ADC measurements, advanced techniques like IVIM and non-Gaussian DWI can provide additional information on tissue microstructure and microvasculature (Fig. 4). These methods show promise in differentiating between malignant and benign breast lesions and predicting metastatic breast cancer [17]. A meta-analysis of 16 studies, examining the diagnostic performance of IVIM-DWI in differentiating breast tumors, found that breast cancers had significantly lower ADC and D values, and higher f values compared to benign lesions. The D value showed the best diagnostic performance (sensitivity 86%, specificity 86%, AUC 0.91) in differentiating breast tumors. IVIM-DWI parameters were found to be superior to ADC alone in distinguishing breast tumors and could further differentiate invasive ductal carcinoma from ductal carcinoma in situ [35]. Diffusion kurtosis imaging (DKI) enhances breast cancer evaluation by quantifying non-Gaussian diffusion behavior at high b-values (>1000 s/mm^2^) through the kurtosis parameter (K), potentially improving differentiation between malignant and benign lesions and providing insights into tumor characteristics beyond standard diffusion-weighted imaging, which typically uses b-values up to 1000 s/mm^2^ [36]. Recent research suggests that DKI parameters, such as higher K, may have prognostic value in breast cancer, with associations found between these metrics and shorter distant disease-free survival [37]. These advanced diffusion techniques show potential for improving diagnosis, treatment monitoring, and outcome prediction in breast cancer management.

## Conclusion

In conclusion, breast DWI has undergone remarkable advancements, offering improved capabilities in detecting, characterizing, and differentiating breast lesions. The ongoing standardization efforts led by organizations like EUSOBI, DWIST, and QIBA are pivotal in harmonizing imaging protocols and enhancing diagnostic accuracy. As these efforts progress, they will play a crucial role in facilitating the widespread adoption of these imaging techniques and ensuring their reliability in clinical practice. The continued refinement of standardization protocols is essential for maximizing the potential of breast DWI in improving patient care and outcomes.
